# Bovine coronavirus presence in domestic bovine and antelopes sub-Saharan Africa: evidence from Namibia

**DOI:** 10.1186/s12917-025-04625-y

**Published:** 2025-03-15

**Authors:** Umberto Molini, Lauren M. Coetzee, Maria Y. Hemberger, Mark Jago, Siegfried Khaiseb, Kalihulu Shapwa, Alessio Lorusso, Giovanni Cattoli, William G. Dundon, Giovanni Franzo

**Affiliations:** 1 Istituto Zooprofilattico Sperimentale dell’Abruzzo e del Molise, Teramo, 64100 Italy; 2 Central Veterinary Laboratory (CVL), 24 Goethe Street, Private Bag 18137, Windhoek, Namibia; 3 Department of Veterinary Medicine, University of Teramo, SP18 Piano d’Accio, Teramo, 64100 Italy; 4 School of Veterinary Medicine, Faculty of Health Sciences and Veterinary Medicine, University of Namibia, Neudamm Campus, Private Bag 13301, Windhoek, Namibia; 5 Meat Corporation of Namibia Ltd. (MEATCO), Northern Industrial Area, P.O. Box 3881, Windhoek, Namibia; 6 Istituto Zooprofilattico Sperimentale delle Venezie, Padua, 35020 Italy; 7 Animal Production and Health Laboratory, Animal Production and Health Section, Department of Nuclear Sciences and Applications, Joint FAO/IAEA Division, International Atomic Energy Agency, PO Box 100, Vienna, 1400 Austria; 8 Department of Animal Medicine, Production and Health, University of Padova, viale dell’Università 16, Legnaro, PD 35020 Italy

**Keywords:** Namibia, Bovine, Coronavirus, Phylogeography

## Abstract

**Background:**

Bovine coronavirus (BoCV) causes significant economic losses to cattle farming due to mortality in calves, reduced growth performances and milk production in feedlots and dairy cattle. Worldwide distribution of BoCV has been demonstrated, although knowledge of its epidemiology in Africa, especially in the sub-Saharan region, is limited.

**Results:**

In the present study, a total of 208 swab samples of wild ruminants and 435 bovines from different regions of Namibia were obtained and tested by a BoCV-specific qRT-PCR. Twenty-six bovine samples tested positive [26/435 (5.98%; 95CI: 3.94-8.64%)] while, among the wild ruminants, only Greater Kudu (*Tragelaphus strepsiceros*) were shown to be positive [13/52 (25.00%; 95CI: 14.03-38.95%)] of which 8 showed clinical signs. Analysis of partial nucleoprotein and spike protein gene sequences and comparison with international reference sequences demonstrated the existence of a unique Namibian clade, resulting from a single introduction event around 2010 followed by local evolution. Although the introduction source remains unknown, contact between bovine and wild animals appears likely.

**Conclusions:**

The present study represents the first report of BoCV circulation in southern Africa, which showed a relatively high frequency and the ability of persisting and evolving locally in the absence of further foreign introductions. The implications for disease spread among domestic bovines and the potential impact on wildlife should encourage broader investigations on BoCV involving other African countries. Moreover, the Greater Kudu’s susceptibility to BoCV infection was also proven, further highlighting the host plasticity of this virus.

## Background

Bovine coronavirus (BoCV) is a positive sense RNA virus, belonging to the family *Coronaviridae*, genus *Betacoronavirus* subgenus *Embecovirus* [1]. Together with other viruses of human and veterinary interest (i.e. human coronavirus OC43, coronavirus HKU23, canine respiratory coronavirus, equine coronavirus, porcine hemagglutinating encephalomyelitis virus and Yak coronavirus), BoCV is classified in the species *Betacoronavirus 1* and because of their close antigenic and genetic relatedness, are considered host-range variants rather than separate species [2, 3]. The virus is characterised by a genome of approximatively 31 Kb; about two-thirds of genomic RNA is occupied by ORF1 which codes for the non-structural proteins pp1a and, through ribosomal frameshifting, pp1ab. These are further processed into the viral polymerase (RdRp) and other non-structural proteins involved in RNA synthesis and cell interaction. Structural proteins are expressed as subgenomic RNAs. ORF3, 4, 8, 9 and 10 encode a hemagglutinin–esterase protein (HE), a spike glycoprotein (S), a small membrane protein (SE), a membrane protein (M), and a nucleocapsid protein (N), respectively. Other ORFs encode additional NSPs, such as 32 kDa and 12 kDa, whose functions have been less investigated [1, 4]. Similar to other coronaviruses, the S protein of BoCV has been well studied and is used in molecular epidemiological analyses. It is involved in viral attachment, conditioning viral tropism, and it is the main target of the host immune response [5]. The S protein is post-translationally cleaved in an S1 and S2 subunit. The S1 is involved in viral attachment and is thus the main determinant of viral tropism, while the S2 anchors the protein to the viral envelope and is implicated in the fusion to the host cell [5]. More specifically, the N-terminal domain (NTD) of the S1 is involved in the attachment to the cell surface glycans while the C-terminal domain (CTD) binds to the host receptor. Host receptor engagement destabilizes the S-trimer, exposing the cleavage site between S1 and S2 subunits to initiate S2-mediated membrane fusion and viral entry. Although limited data are available, it has been speculated that BoCV also utilizes the two-receptor binding motif (RBM) system, using 5-N-acetyl-9-O-acetylneuraminic acid (Neu5, 9Ac2) as glycan attachment molecules and HLA-I as a protein attachment receptor recognized by S1-CTD [6]. It has been proposed that the two RMB system might assist the ability of coronaviruses to cross host species barriers with NTD glycan binding allowing a minimum binding and infectivity mediated by sialic acids while CTD can evolve gaining adaptative mutations that optimize the binding to a new host receptor. The role of the hemagglutinin–esterase protein in broadening host tropism by enabling the virus to bind to different cell types, has also been proposed [7].

The host plasticity of BoCV has been shown in several experimental and epidemiological studies, where it has been detected in a large number of wild and domestic ruminant species (see [2] for review).

In cattle, BoCV leads to considerable financial losses because of calf deaths and lowered growth rates and milk yield in adult cattle raised for beef and dairy production. It is considered a major cause of calf diarrhea during the first weeks of age in both dairy and beef calves and winter dysentery in adult cattle. It has also been associated with respiratory signs in animals of all ages, and together with other viruses and bacteria, BoCV is part of the bovine respiratory disease complex (BRDC) [1, 6, 8, 9].

Although not as common, BoCV and BoCV-like coronavirus have been reported in other domestic and wild species in the presence of clinical signs [2, 3, 10–13] . To date, a clear link between specific viral mutations and enteric/respiratory tract tropism has not been identified for BoCV [14, 15]. As BoCV has been reported worldwide, enough sequence data has been generated to allow for the identification of clear geographic clustering which distinguishes European-origin from American–Asian origin viruses[6]. Currently, only two reports on the presence of BoCV in Africa are available, one from Algeria/Ethiopia and one from Ghana [16–18]. There are no reports from the Sub-Saharan region.

The present study aimed to fill this knowledge gap by testing domestic cattle and several wild ruminants species in Namibia using a specific real-time RT-PCR assay. Genetic characterization through partial sequencing of the S and N protein-coding ORFs was also performed on positive samples.

## Results

### BoCV detection

**Fig. 1:**
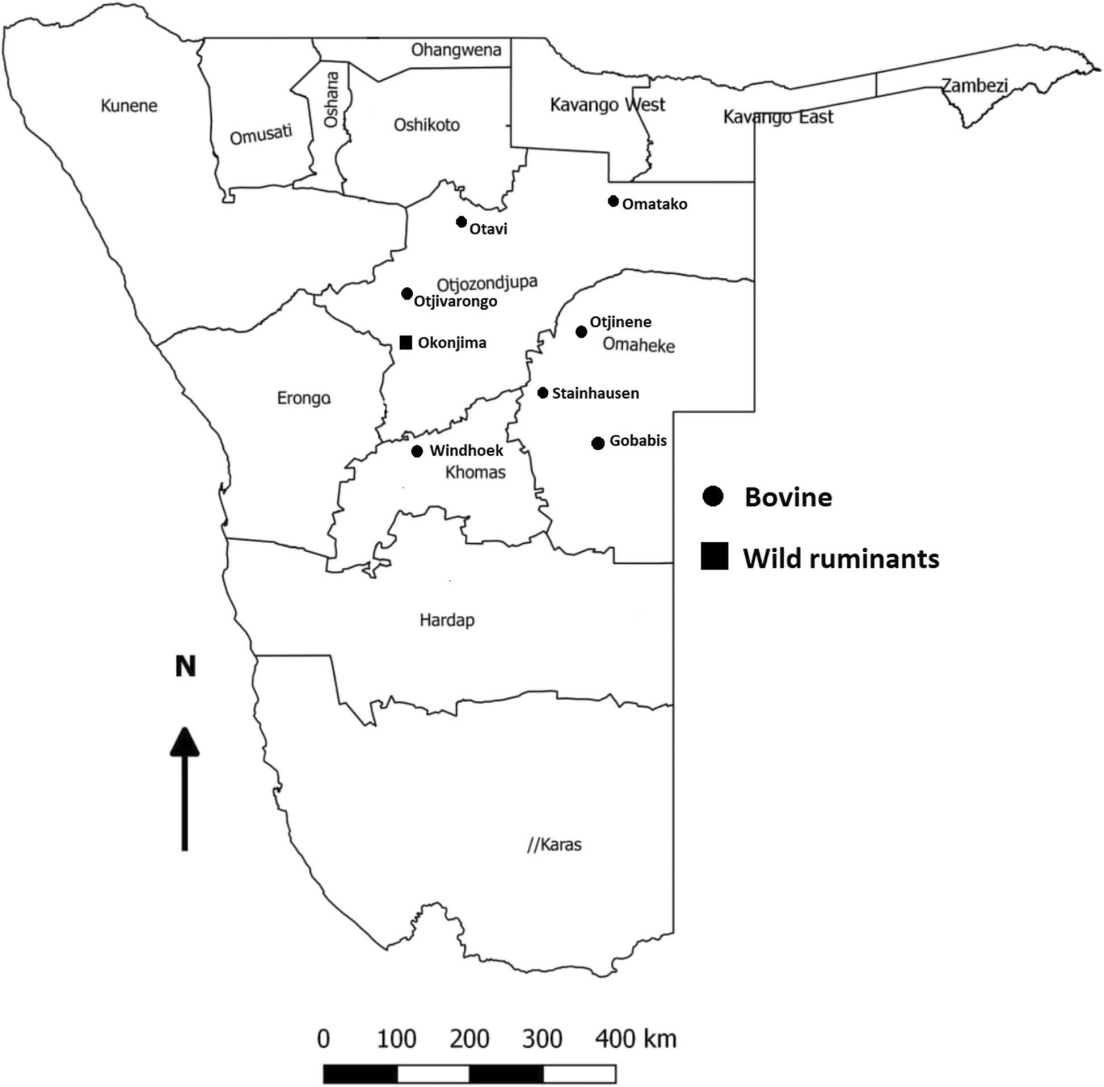
Map of Namibia indicating the regions where BoCV was detected in cattle and wild ruminants from September 2019 to October 2022. The filled circles and squares represent cattle and wild ruminants, respectively. The figure was produced by Central Veterinary Laboratory (CVL), Windhoek, Namibia

Out of the 435 bovine swabs, 26 samples tested positive (5.98%; 95CI: 3.94–8.64%) by qRT-PCR (Figure 1 and Table 1) with a Ct that ranged between 26.97 and 38.36. The presence of BoCV was reported in most but not all of the regions of Namibia investigated, with variable frequency (Table 1). None of the bovines involved in the study showed clinical signs related to BoCV infection during ante-mortem inspection.

**Table 1:** Number of tested and positive bovine samples classified according to the collection district and region

**District**	**Region**	**Samples**	**Positive samples**
Gobabis	Omaheke	99	6
Otjinene		12	1
Stainhausen		24	1
Kamanjab	Kunene	19	0
Outjo		26	0
Mariental	Hardap	26	0
Omatako	Otjozondjupa	32	9
Okahandja		24	0
Otjivarongo		23	1
Otavi		33	7
Grootfontain		15	0
Windhoek rural	Khomas	63	1
Keetmanshoop rural	Kharas	6	0
Tsumeb	Oshikoto	9	0
Katima Mulilo	Zambezi	24	0

Of the 208 swab samples from wild ruminants analyzed in this study, only 13/52 (25.00%; 95CI: 14.03–38.95%) samples from Greater Kudu (*Tragelaphus strepsiceros*) tested positive by qRT-PCR with a threshold cycle (Ct) ranging between 22.40 and 28.27. All of the 13 wild ruminants were part of a group used for a vaccine trial, allocated in a wild life facility and monitored 24/7, and came from the same game reserve located in the Otjozondjupa region of Namibia. Eight out of these 13 greater Kudus presented an enteric infection characterized by profuse diarrhea for one week.

### Sequence analysis

Fragments of the N gene were amplified and sequenced from eight of the 26 positive samples while the partial S gene was amplified and sequenced from seven of the 26 positive bovines. N and S gene fragments were obtained for all of the Greater Kudus. All obtained sequences were submitted to GenBank (Acc.Numbers OR161840-OR161879). No evidence of recombination was revealed in the selected region at the set parameters.

**Fig. 2:**
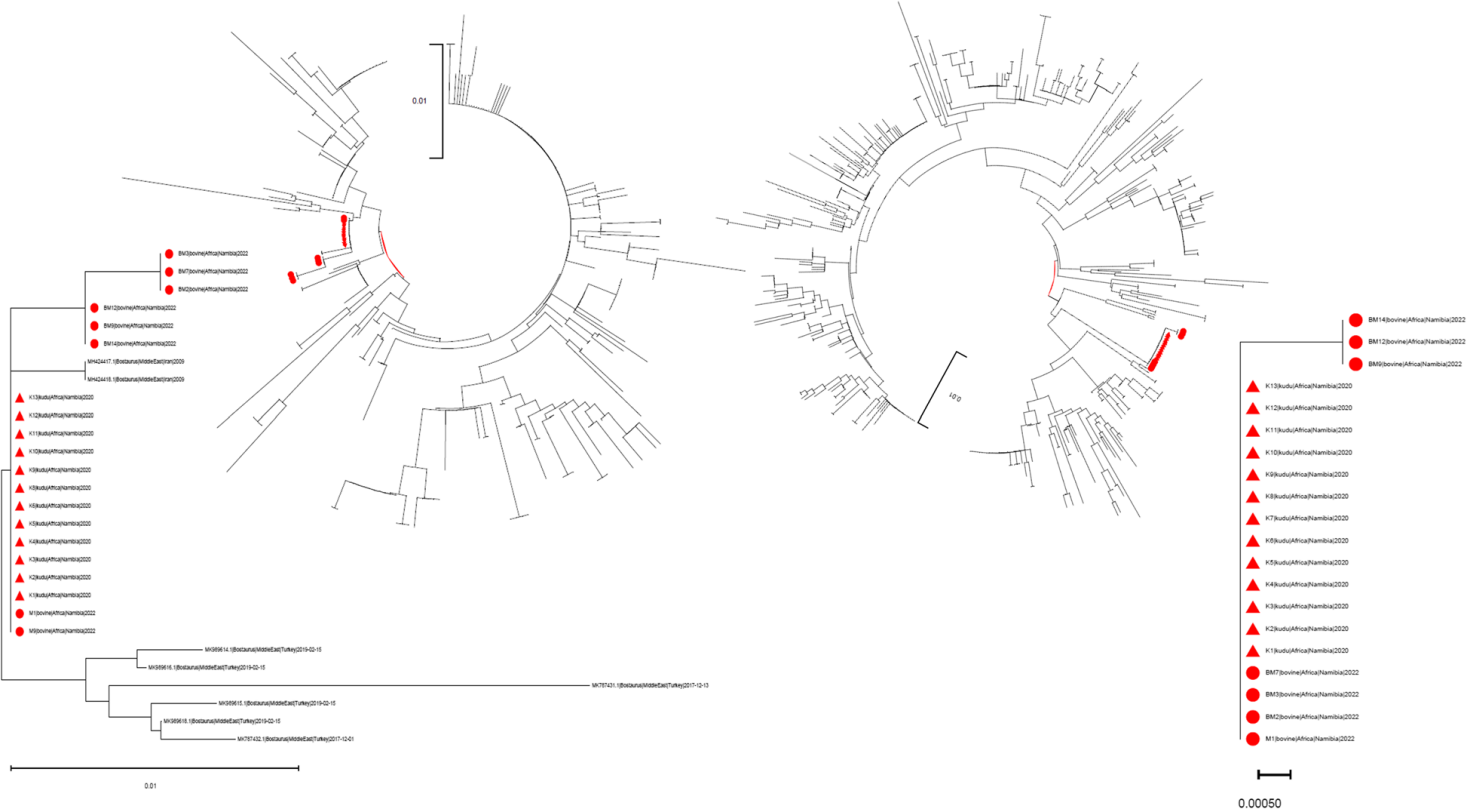
Maximum likelihood phylogenetic tree based on partial N (left) and S (right) sequences. The Namibian strains are highlighted as red circles (bovine) and triangles (Greater Kudus). The specific clades are magnified in the inserts

The Namibian N gene sequences showed a low genetic distance between each other, ranging from 0 and 0.539% and formed a clade with sequences from Iran and Turkey (minimum generic distance *p* = 0.265%) (Fig. 2). All of the sequences from BoCV detected in Greater Kudus were identical to each other and to two bovine strains (i.e. M1|bovine|Africa|Namibia|2022 and M9|bovine|Africa|Namibia|2022).

The Greater Kudu strains has identical S sequence, which were also identical to bovine sequences BM7|bovine|Africa|Namibia|2022, BM3|bovine|Africa|Namibia|2022, BM2|bovine|Africa|Namibia|2022, M1|bovine|Africa|Namibia|2022). The genetic distance among S gene sequences obtained from the bovine samples ranged from 0 to 0.16%. An independent Namibian clade was identified in the S gene phylogenetic tree (Fig. 2). Although geographical clustering was present, some strains collected in different areas were mixed according to both N and S gene-based phylogenetic trees.

**Fig. 3:**
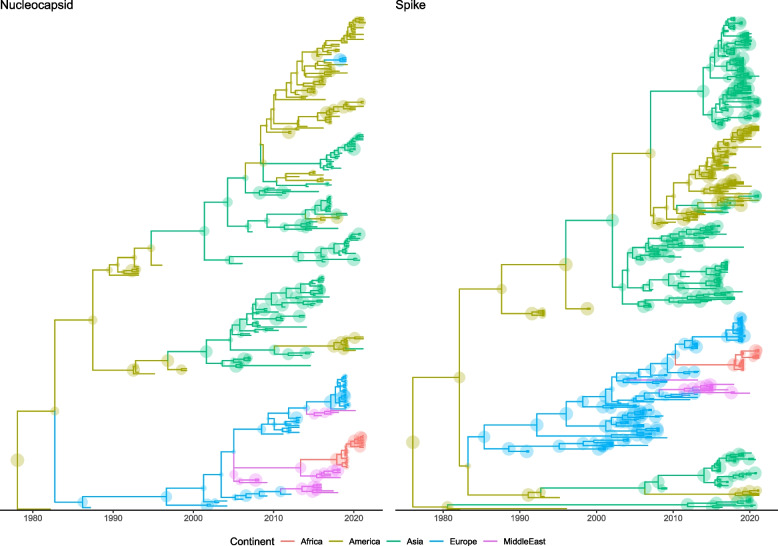
Time-scaled maximum clade credibility tree reconstructed based on the S and N partial gene sequences. The branches are color-coded according to the most likely continent where the ancestral strains were predicted to circulate. Circle size is proportional to the posterior probability of the corresponding node

The serial coalescent analysis based on the partial N gene alignment estimated that Namibian sequences likely originated around 2013 [95HPD: 2008.01–2016.91] from Middle Eastern (i.e. Iran and Turkey) countries, which in turn were part of a European strains clade (Fig. 3).

A comparable date of introduction was estimated using the S gene (i.e. 2011 [95HPD: 2009.04–2012.68], followed by independent evolution. The most likely viral introduction source was predicted from Europe, which was different from the N gene prediction (Fig. 3). However, the N and S sequences available from the Middle East were not from the same samples.

## Discussion

Together with other enteric and respiratory pathogens, BoCV is responsible for significant economic losses to the bovine farming sector worldwide [1]. The impact can be particularly severe in low-income areas where bovine farming has an important role in human subsistence. The present study demonstrates BoCV positive rates of about 5% in the Namibian bovine population, albeit with regional variations. Comparison with other reports is challenging considering the extremely heterogeneous sampling approaches and populations evaluated in different studies (e.g. animal and farming type, presence of clinical signs, etc.) [6]. Nevertheless, such a positivity rate can be considered quite high since adult, asymptomatic animals were considered. The occurrence of BoCV infection in healthy subjects is not uncommon, where viral detection has been reported to range between 0–46% [6]. Several contributing factors are necessary for overt disease emergence. Bovine breed, animal density, co-infections, transportation and housing related stresses, climate and other environmental variables have been implicated. In Namibia, two types of farming are practiced, namely commercial and subsistence farming. Subsistence agriculture is primarily limited to the communal lands in the densely populated northern region of the country, where free-ranging cattle are common.. There are about 4,000 commercial farms in Namibia which contribute to roughly 63.44% of the total Namibian bovine export market. Cattle grazing is predominant in the central and northern regions of Namibia (Meatboard annual report 2020–2021, https://nammic.com.na/annual-reports/). In both types of farming models, bovines are typically raised at pasture; therefore several of the above-mentioned risk factors for disease emergence might not be present. Similar conclusions were drawn in a study performed in Ghana, where an even lower viral circulation in free-roaming cattle was reported [17].

The analysis of the partial N and S genes demonstrated the epidemiological isolation of Namibia from other countries and that all Namibian strains most likely originated from a single introduction event that occurred around 2010 and evolved independently since then. The estimated area of origin was the Middle East or Europe, depending on the dataset. However, it must be stressed that sequence availability and representativeness, especially from Africa was extremely poor. Therefore, several alternative links might have been missed. No live bovines from neighboring countries have been introduced officially in the last years while most of the genetic material is imported into Namibia from Europe although bovine semen is not considered a significant transmission source of BoCV[19]. The contribution of wild animals, crossing national borders without control, cannot be excluded. Several wild species have been shown to be positive for BoCV and BoCV-like strains, both in symptomatic and asymptomatic animals [2]. In the present study, only Greater Kudu tested positive for BoCV. The lack of positivity in the other wild ruminants is not unexpected since the excretion phase of the virus, although prolonged can be missed by direct viral detection methods [20–22]. Further studies, based on serological testing might be of help in further investigating this topic.

The positive Greater Kudus were from a game reserve where no domestic animals were present. Contact with domesticated ruminants, including pasturing cattle, is prevented by fences. More in detail, the 13 test-positive individuals were part of a cohort of Greater Kudu which were selected for an experimental study (UNAM ethics clearance certificate n: AREC/023/2020) and transported to the wildlife isolation research facilities at the Neudamm campus using a game truck that is understood to have not been previously used for the movement of cattle. After approximately two weeks in the experimental facilities, several subjects developed diarrhea. Because of the strict biosecurity measures implemented during the whole process, it is believed that the infection may have originated within the reserve. Viral circulation in the Greater Kudu population or other wild animals is thus a possibility. Susceptibility of antelopes to BoCV, including members of the same genus [i.e. sitatunga (*Tragelaphus spekei*), nyala (*Tragelaphus angasii*)] [23, 24] has previously been reported and can be reasonably extended to the present scenario.

The sequences obtained from the Greater Kudu samples were identical to bovine ones, indicating the exchange of strains between the two populations. Moreover, the area where the game reserve is located was chacteriszd by a high rate of BoCV detection (Table 1). How the virus may have been introduced into the game reserve despite the protective fences requires further investigation. Fences might not effectively protect from pathogen introduction through indirect contacts mediated by passive vectors, neighboring environmental contamination, or even personnel and their cars/trucks. The directionality of the viral flux and whether it was bovine-to-Kudus, Kudus-to-bovine*,* or bidirectional remains to be established. If BoCV was the cause of the diarrhoea in Greater Kudus, the relatively long lag phase between animal introduction into the isolation facilities and clinical signs development was unexpected since a 1–7 day incubation is usually seen [25]. Because the sequences from the Greater Kudus were identical to each other it is feasible that a single animal infection followed by viral transmission to other animals due to close contact in the experimental facilities occurred. Therefore, an initially asymptomatic subject could have infected the others, which then developed clinical signs potentially facilitated by stress due to transportation and housing conditions. Alternatively, the incubation phase of BoCV, might differ between bovine and Kudus.

## Conclusion

The present study represents the first report describing the Greater Kudu’s susceptibility to BoCV infection, further highlighting the host plasticity of this virus. Moreover, the epidemiology of BoCV in southern Africa is herein investigated, which showed a relatively high circulation and the ability of the virus to persist and evolve locally in the absence of further foreign introductions. Despite the absence of clinical signs in domestic bovines, the relevance of the infection should not be ignored since other domestic animal species might be more severely affected. Moreover, the susceptibility of wild species and the occurrence of clinical signs testify that viral circulation and strain exchange might represent a severe threat to wild species, including endangered ones. Transmission of BoCV from cattle to other species and *vice versa* could determine the recurrent emergence of new epidemics and potentially favor viral evolution. Additionally, the free movement of wild animals across regional and national borders could be a factor further favoring viral dispersal and strain mixing.

Consequently, additional research is needed to gain a deeper insight into the molecular epidemiology of BoCV, particularly in Africa where interaction between various animal populations (both wild and domestic) is common. This would also include exploring the trends and factors influencing the spread of the virus across different countries.

## Material and methods

### Sample collection, processing and testing

The study included 208 wild ruminant nasal swabs obtained during the period September 2019– August 2020 from two national parks, two private game reserves, and one commercial farm around Namibia (Table 2). Nasal swabs of 435 bovines from 120 commercial farms across 15 districts in 8 Namibian regions, collected during 2022 from four commercial abattoirs, were also included in the study (Table 1). Sterile dry nasal swabs were used for each animal. The bovines were tested randomly after slaughter (following pre- and post-mortem examination), while samples from the wild animals were collected during routine game monitoring activities after sedation. All swabs, after collection, were stored at +4°C and sent to the laboratory for BoCV screening. The Study was performed under the approval of UNAM ethics commission: UNAM ethics clearance certificate number: AREC/023/2020. All methods were performed in accordance with the relative ethical guidelines and University and National regulations. In the laboratory, each swab was suspended in 500 µl of sterile PBS and vortexed for 2 minutes at the maximum speed. Three hundred µl of the suspension were used for RNA extraction. Total genomic RNA was extracted using High Pure Viral Nucleic Acid Kit (Hoffman-La Roche, Switzerland) with an elution volume of 100 μl following the manufacturer's instructions. RNA extracts were screened using an in-house real-time RT-PCR (qRT-PCR) assay with specific primers and probe designed for the N gene of BCoV (Table 3). Briefly, qPCR was performed on a C1000 Bio-Rad thermocycler (Bio-Rad Hercules, CA, USA) with Oasig lyophilized OneStep qRT-PCR MasterMix (Genesig Primerdesign Ltd, Camberley, UK) with the following thermal profile: 50°C for 3 min, 95°C for 30 s, followed by 40 cycles at 95°C for 3 s and 60°C for 12 s. A positive control provided by the Istituto Zooprofilattico Sperimentale dell’Abruzzo e Molise (IZS) and a negative control (free DNA/RNA water) were used.

**Table 2:** Number of tested and positive wild ruminant samples, classified according to species

**Species**	**Samples**	**Positive samples**
Red hartebeest (*Alcelaphus buselaphus caama*)	26	0
Greater kudu (*Tragelaphus strepsiceros*)	52	13
Oryx (*Oryx gazella*)	37	0
African buffalo (*Syncerus caffer caffer*)	24	0
Springbok (*Antidorcas marsupialis*)	29	0
Eland (*Taurotragus oryx*)	15	0
Blue wildebeest (*Connochaetes taurinus*)	11	0
Impala (*Aepyceros melampus*)	7	0
Sable antelope (*Hippotragus niger*)	5	0
Waterbuck (*Kobus ellipsiprymnus*)	2	0

**Table 3:** Primers and probes used in this study

**Primers and probe**	**Sequence (5’−3’)**	**Product length (bp)**
qBoCV_F1	TCCACAGTTCCCCATTCTTG	85bp
qBoCV_R1	TCTGCACTTTGGCCAACTCT	
qBoCV_Probe	FAM-ACTCGCACCCACAGCTGGTG-BHQ	
BCV N F	GCCGATCAGTCCGACCAATC	407bp
BCV N R	AGAATGTCAGCCGGGGTAT	
S S1A F	ATGTTTTTGATACTTTTAATT	654bp
S S1A R	AGTACCACCTTCTTGATAAA	

For molecular characterization, the RNA of the samples that tested positive for BCoV were further amplified using two different sets of primers targeting a 654 bp fragment of the S gene (Liu et al., 2006) and a 407 bp fragment of the N gene (Fukuda et al., 2012) (Table 3). All the reactions were performed with the OneStep RT-PCR Kit (Qiagen) with the following thermal profile: reverse transcription at 50° for 30min, initial denaturation at 95 °C for 15 min, 40 cycles of denaturation at 94 °C for 45 s, annealing at 52 °C (for N gene) or 50 °C (for S gene) for 45 s, extension at 72 °C for 1 min followed by final elongation at 72 °C for 10 min. The PCR products were visualized on 2% agarose gel stained with ethidium bromide. The amplicons were purified using a Wizard SV Gel and PCR Clean-Up System (Promega) and sequenced commercially by LGC Genomics (Berlin, Germany).

The results of the diagnostic activity were summarized as detection frequencies with relative confidence intervals, calculated using the Binomial (Clopper-Pearson) 'exact' method in Epitools (https://epitools.ausvet.com.au/).

### Sequence analysis

A complete collection of S and N gene sequences of BoCV overlapping with the sequences obtained in the present study was downloaded from GenBank. Metadata on collection country, host and date were associated with the sequence name when available. Unverified sequences and those of poor quality, including premature stop codons, frameshift mutations, unknown bases or obvious misalignments were removed from the dataset.

Sequences were aligned with the ones obtained in the present study using MAFFT [26]. Evidence of recombination was tested using the Genetic Algorithm for Recombination Detection method (GARD) [27]implemented in Datamonkey [28] and RDP4 [29]. The RDP4 settings for each method were adjusted to account for the dataset features according to the RDP manual recommendations. RDP, GENECONV, Chimaera and 3Seq were used in a preliminary scan while the full set of available methods was used for analysis refinement. Recombination events detected by more than two methods with a significance value lower than 10^−5^ and Bonferroni correction were accepted.

Maximum likelihood (ML) phylogenetic trees were reconstructed using IQTree [30], selecting the substitution model with the lowest Bayesian Information Criterion (BIC).

The potential source and timing of viral introduction, as well as other population parameters, were estimated using the Bayesian serial coalescent approach implemented in BEAST 1.10.0 [31]. The nucleotide substitution model was selected based on the Bayesian Information Criterion (BIC) calculated using JModelTest2 [32], while the molecular clock model was chosen based on Bayesian factor (BF) calculation obtained by estimating the marginal likelihood of the evaluated models using the path sampling (PS) and stepping stones (SS) methods [33]. The non-parametric Skyline [34] model was selected to account for fluctuations in the relative genetic diversity (i.e. effective population size x generation time; Ne x t) over time. Strain migration among continents was reconstructed using the discrete state phylogeographic approach described by Lemey et al. [35] considering each collection continent as a strain trait. All parameters were jointly estimated using a 200 million generation Markov Chain Monte Carlo (MCMC) chain, sampling the population parameters and trees every 20 thousand generations. Run performances were summarized and evaluated using Tracer 1.7 after removing the first 20% of the data as burn-in. Run results were accepted only if the Estimated Sample Size (ESS) was higher than 200 and the mixing and convergence, evaluated by visual inspection of the run’s trace, were adequate. A Maximum Clade Credibility tree (MCC) was obtained summarizing over the tree posterior distribution using the Treeannotator suite of the BEAST package.

## Data Availability

All obtained sequences have been submitted to GenBank (Acc.Numbers OR161840-OR161879) and the accession numbers are provided in the article.
